# Systematic Review and Meta-Analysis of Clinically Relevant Executive Functions Tests Performance after COVID-19

**DOI:** 10.1155/2023/1094267

**Published:** 2023-02-09

**Authors:** Boris B. Velichkovsky, Anna Y. Razvaliaeva, Alena A. Khlebnikova, Piruza A. Manukyan, Vladimir N. Kasatkin, Artem V. Barmin

**Affiliations:** ^1^Research Institute for Brain Development and Peak Performance, Peoples' Friendship University of Russia, Moscow 117198, Russia; ^2^Lomonosov Moscow State University, Moscow 125009, Russia; ^3^Institute of Psychology, Russian Academy of Sciences, Moscow 129366, Russia; ^4^Cognitive Foundations of Communication Laboratory, Moscow State Linguistic University, Moscow 119034, Russia

## Abstract

It is widely known that COVID-19 has a number of prolonged effects on general health, wellbeing, and cognitive functioning. However, studies using differentiated performance measures of cognitive functions are still not widely spread making it hard to assess the exact functions that get impaired. Taking into account the similarities between post-COVID ‘brain fog' and chemofog, we hypothesized that executive functions (EF) would be impaired. Literature search yielded six studies with 14 effect sizes of interest; pooled effect size was small to medium (*d* = −0.35). Combined with a narrative synthesis of six studies without a comparison group, these results show that EF get impaired after COVID-19; although, in most cases the impairment is transient and does not seem to be severe. These results specify the picture of ‘brain fog' and may help to discover its mechanisms and ways of helping people with long COVID.

## 1. Introduction

Since it emerged in December 2019, COVID-19 has caused a global healthcare crisis and posed a number of problems. Concerns about its prolonged impact on survivors have arisen, corroborated by their frequent reports of fatigue, cognitive impairments (also dubbed ‘brain fog'), shortness of breath, sleep disturbances, depression, anxiety and post-traumatic stress disorder, impaired or distorted sense of smell and taste, muscle pain, and headaches [1, 2]. These symptoms have a negative impact on the quality of life, mental health, and employment, can last for months after recovery and emerge even in non-hospitalized or asymptomatic patients [1, 3]. Prolonged effect of COVID-19 could also be observed in escalation in severe mental illnesses, such as psychosis [4].

It is hypothesized that cognitive impairment after COVID-19 is linked to the impact of the disease on neurological, immune, and vascular systems [5, 6]. Patients in more severe condition (e.g., requiring ventilation) may develop multiple diffuse lesions in the white matter [7]. Global cognitive impairment after COVID-19 has also been linked to volume changes in the white matter, especially in the frontal lobes [8]. These and other neurological complications may be contributed to the effects of neuroinflammation, neurotoxicity, or hypoxia [5]. It is worth noting that hypoxemia can be considered as the cause of psychiatric long-term consequences of COVID-19, such as an escalation in psychosis [9]. According to the recent study [10] among the possible causes of the so-called ‘Cognitive COVID' (acute changes during COVID-19 and after it) are systemic inflammation, multisystem inflammatory syndrome and ventilation, psychological strain caused by pandemic and lockdowns, direct neurotropism, and subsequent brain damage. However, the exact mechanisms behind the cognitive sequelae have not been discovered yet, and one of the possible reasons for that is a lack of studies focusing on specific cognitive functions. Executive functions (EF) could be a good focus point because their impairments are associated with conditions closely resembling post-COVID ‘brain fog'.

EF are broadly defined as a prerequisite for performing complex tasks where automatic behaviors are maladaptive, for example, when rules change, new distractors emerge or risks require assessment. Key EF include inhibition, cognitive flexibility, selective attention, and working memory; they are closely related to fluid intelligence, problem solving and decision-making skills, and quality of life [11]. A series of studies by Miyake and Friedman illustrates that EF contain common components, but shifting and updating have specificity that cannot be explained by the common factor [12]. The latent common factor uniting EF can explain the methodological problem with validity and ‘task purity', when measures designed to assess a single EF can be related to other complex executive and non-executive mental processes [13]. Specificity, on the other hand, can explain the use of different strategies in solving common problems [12, 14].

Evidence points to disruptions in EF due to somatic illnesses, especially in chemobrain, a condition prevalent in cancer patients after chemotherapy and characterized by cognitive decline and difficulties in everyday tasks [15]. Cancer survivors show significantly lower scores on inhibition, switching, and planning tasks, compared with normative scores [16]. These changes are related to decreases in white matter volume and functional changes [17]. Links between EF (visuospatial learning, lexical access, and set shifting), cytokines, and helper and cytotoxic T-cell count have also been discovered in chronic fatigue syndrome research [18]. Inhibition, working memory, and set shifting are also impaired in adolescents with type 1 diabetes [19]. Given that neuroinflammation plays an important role both in chemobrain and brain fog [20], we could expect similar deficits in EF in people with post-COVID syndrome.

Mental distress also has a negative impact on EF. Acute stress leads to worsening accuracy in task management, planning, and coding measures [21], as well as working memory and task flexibility impairments [22]. The effect of acute stress on inhibition is not straightforward: while it impairs cognitive inhibition, response inhibition slightly increases, which signifies greater ability to control one's behavior [22]. Inhibitory control, planning, flexibility, decision-making, and sustained attention scores are lower in students with depression and anxiety symptoms [23]. Given the high prevalence of stress-related mental disorders in COVID-19 survivors and their correlation with cognitive deficits [24], this is another factor that could contribute to deficits in EF.

The current study was carried out in order to determine to what extent EF get impaired after recovery from COVID-19. The research question was formulated in accordance with population–exposure–comparator–outcome (PECO) formula as follows: How do EF change in people after COVID-19 in comparison with healthy controls?

## 2. Materials and Methods

### 2.1. Eligibility Criteria

Studies were included if they: (a) described people with confirmed COVID-19, (b) and compared them with healthy controls on (c) objective cognitive measures of EF. Studies were excluded if they: (a) used global measures of cognitive functioning or self-report measures, did not assess particular EF and (b) focused on patients with other chronic illnesses or cognitive dysfunction preceding COVID-19. Studies without a healthy control group were excluded from meta-analysis, but if they used objective tests of EF, they were included in the narrative review.

### 2.2. Information Sources

We conducted searches in Scopus, Web of Science, and PubMed citation databases. Medical and psychological preprint databases (PsyArXiv, medRxiv, bioRxiv, and Research Square) were included in accordance with the guidelines on decreasing publication bias. The latter also helped to gain more papers given the novelty of the field and the stringency of the eligibility criteria.

### 2.3. Search Strategy and Selection Process

Initial searches were conducted in November–December 2021. Keywords related to SARS-CoV-2, cognitive deficit, and EF were used. In July–August 2022 citation bases were searched again in an attempt to discover more papers that fit eligibility criteria.

Search results were uploaded to the CADIMA online tool for systematic reviews [25]. After duplicate removal, titles and abstracts were screened independently by three reviewers (AKh, PM, and AR).

### 2.4. Data Collection

The following data items were collected from the studies:

(i) Study information: authors and year of publication.

(ii) Demographic information: number of participants, sex, age, country of residence, and education.

(iii) COVID-specific information: time since illness and illness severity (number of hospitalized patients in COVID+ group and number of those who required intensive care and/or ventilation).

(iv) Results of measuring EF: means and standard deviations, names of tests, and/or scales.

Working memory and attention measures were not included in the current meta-analysis as they were analyzed and described in detail elsewhere [26].

### 2.5. Risk of Bias Assessment

To assess study quality we used the Johanna Briggs Institute Critical Appraisal Checklist for analytical cross sectional studies [27]. Studies were analyzed independently by three reviewers; consensus on any points of disagreement was reached during a group discussion.

### 2.6. Pooling Effect Sizes Scales

To measure effect sizes, standardized mean differences (Cohen's *d*) were calculated on the basis of group means, standard deviations, and sample sizes reported in the original studies. If the study reported medians and ranges [28], they were converted to means and standard deviations according to formulas provided by Hozo et al. [29].

We used random-effect models to pool effect sizes (as opposed to fixed-effect models that operate on the assumption of a single true effect size). This approach is effective when the original studies employ different samples (e.g., from different countries), data gathering techniques and measures; that is, when the assumption of a homogeneous population cannot be upheld [30].

Given that a lot of studies use more than one test to measure EF and report numerous effect sizes, we decided to use a multilevel approach when pooling them. It can reduce researcher bias stemming from picking out a single effect from each study.

Heterogeneity was expected to be high based on variability in measures, study designs, and sample characteristics. To measure it *I*^2^ statistic was used, and the value of 75% or more was considered indicative of high heterogeneity.

Sensitivity analysis was performed using leave-one-out approach, when the model was retested after removing a single observed outcome at a time. This approach can show which study contributed to the pooled effect size the most. Publication bias was tested by Egger's regression test. In case it could not be calculated directly we modelled it by including standard errors as a moderator in the model.

Data were analyzed in the R version 4.1.2 [31]. The following packages were used:

(i) esc ver. 0.5.1 [32]—to compute effect sizes (Cohen's *d*);

(ii) metaphor ver. 3.0-2 [33]—to pool effect sizes, perform sensitivity analysis, assess publication bias, and draw plots.

## 3. Results

### 3.1. Study Selection

**Figure 1:**
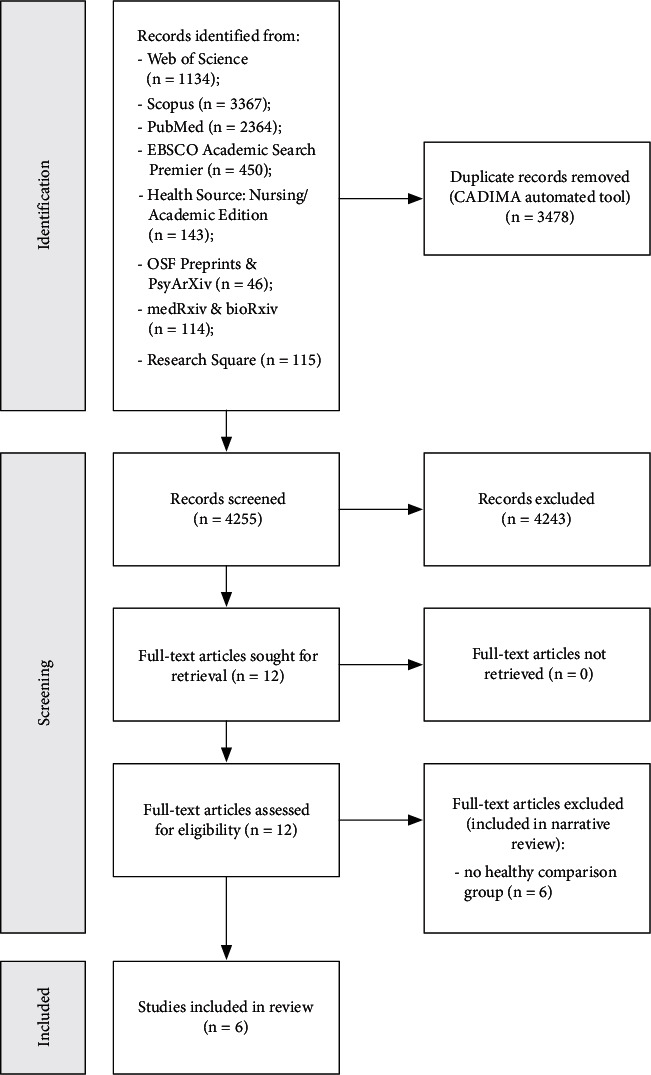
PRISMA flowchart for study identification and selection.

The results of the selection process are shown in Figure 1. Twelve relevant papers were identified as a result of the search. Six studies were only included in the narrative synthesis because they reported EF scores only in post-COVID group. Thus, six papers were included in the meta-analysis. Participants, measures, and overall bias scores for the studies are shown in Table 1. In case of Wild et al. [34] patients with acute COVID were included in the sample, which made it necessary to recalculate overall effect sizes excluding this group of participants.

All studies were of acceptable quality. There were several concerns raised in quality assessment. Online-based study design applied by Wild et al. [34] and Zhao et al. [35] could lead to self-selection of participants who did not have laboratory-confirmed COVID-19, but self-diagnosed. These studies also did not report the country of residence of the sample making it harder to determine possible cultural input into the results. Guo et al. [36] included participants without laboratory-confirmed COVID-19 and did not report illness severity (% of patients who had required hospitalization). Mattioli et al. included participants as early as 12 days after recovery [28].

### 3.2. Measures Used in the Studies

The following measures of EF were used in the studies: Trail Making Task-B, Stroop Color-Word Interference test, Tower of London task, and Wisconsin Card Sorting Task. The tasks are described in Table 2. Although all these tasks involve multiple EF, Stroop Color-Word Interference test is considered a measure of inhibition and Tower of London measures planning and reasoning. Wisconsin Card Sorting Task and Trail Making Task-B are used in brain injury testing (the former in particular is considered a frontal lobe task) and measure set shifting, task switching, and updating.

### 3.3. Results of Individual Studies

A total of 14 effect sizes were collected from the studies (Table 3). Effect sizes (Cohen's *d*) ranged from −0.80 to 0.10; a half of them were statistically significant.

Studies not included in meta-analysis also reported a decrease in EF in post-COVID patients. Hampshire et al. [37] discovered larger decreases in planning (measured by the Tower of London task) for people who had been hospitalized and put on ventilation compared with those who did not exhibit or had only mild respiratory symptoms. Effect size for this subgroup of patients was small to medium and ranged from −0.36 for all cases (including those who suspected COVID) to −0.42 for those with a positive COVID-19 test [37].

Silva et al. [38] reported an abnormally low performance on the Trail Making Task-B for 40% of the sample of 87 non-hospitalized individuals. They also found a correlation (*r* = 0.30) between performance on this task and white matter impairments in inferior longitudinal fasciculus (an association tract that connects occipital and temporal lobes).

Miskowiak et al. [39] compared patients' (*n* = 29) performance on Trail Making Task-B to sex-, age-, and education-adjusted norms revealing a large significant decrease in cognitive flexibility (*d* = −0.81). In another study by the same group of authors hospitalized patients (*n* = 29) performed worse on the same task than non-hospitalized ones (*n* = 19); *d* = −0.78 [40].

Larsson et al. [41] found no differences between ICU-admitted patients and those who had less severe symptoms on Trail Making Task-B (*d* = −0.06); however, older participants (65+ years old) performed this task significantly slower than younger ones (*d* = −0.54).

Surprisingly, comparing scores of patients after COVID-19 (*n* = 31, approximately 6 months after positive test) with population norms on Color-Word Interference task and Trail Making Task-B, Dressing et al. [42] did not find any participants with scores below mean value.

### 3.4. Effect Sizes Synthesis

**Figure 2:**
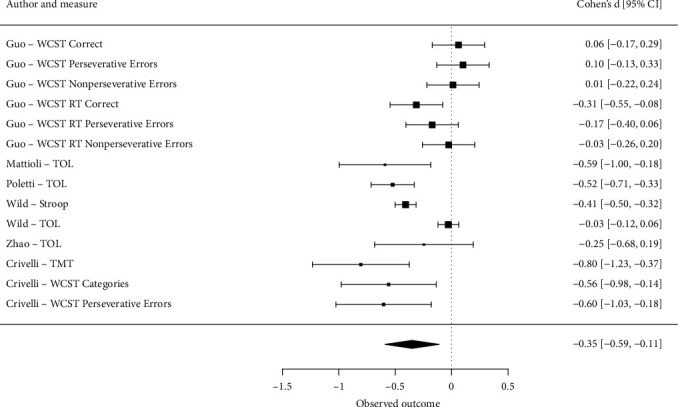
Forest plot for the multilevel random effects model. WCST—Wisconsin Card Sorting Test; RT—reaction times; TOL—Tower of London; Stroop—Stroop Color and Word Test; TMT – Trail Making Task -B. Squares represent original effect sizes; their area corresponds to the weight of the study (number of participants). Black diamond at the bottom is the pooled effect size.

The pooled standardized mean difference between post-COVID-19 participants and healthy controls was −0.35; 95% CI [−0.59, −0.11], *p* < 0.05.  Figure 2 presents the forest plot for the model.

Variance testing on different levels showed that Level 3, or between-study heterogeneity (*I*^2^ = 58.79) was larger than Level 2, or within-study heterogeneity (*I*^2^ = 27.97). Sampling error (Level 1) variance was 13.24. Overall heterogeneity was large (*I*^2^ = 86.76), possibly due to different measures used in the studies, and different study designs (online and offline) and samples (clinical samples recruited in clinic or self-recruiting participants in online studies who self-reported their symptoms).

Sensitivity analysis was carried out in two ways. First, effect sizes were removed one by one. Pooled effect size varied from −0.40 to −0.32; the largest value was reached when the results of Tower of London test from Wild et al. [34] were removed. The removal of the same test from Poletti et al. [43] on the contrary impacted the value in the opposite direction. A more traditional leave-one-out analysis was also performed when studies were removed on the whole. In this case pooled effect size varied from −0.43 (after six effect sizes from Guo et al. [36] were removed) to −.26 (after three effect sizes from Crivelli et al. [44] were removed).

Publication bias could not be assessed directly (due to the use of multilevel modeling). The model was rerun, and standard error was included as a moderator. According to the results publication bias can be considered insignificant (*b* = −1.84, 95% CI [−5.28, 1.59], SE = 1.58, *t* = −1.17, *p* = .27). However, they should be interpreted with caution due to the interdependencies between effect sizes coming from the same study.

## 4. Discussion

People who recover from COVID-19 often report cognitive dysfunction as one of the long-lasting symptoms. Although memory difficulties and poor attention and concentration are more often cited in reference to these symptoms, objective measures show that EF (such as inhibition, updating, and set shifting) also get impaired. Effects range from small to large, and generally decreases in performance are related to advanced age and illness severity. These results corroborate earlier systematic analyses [45, 46] and further validate their points by pooling effect sizes from studies with healthy control groups.

Current findings can be translated into clinical practice through diagnostics of cognitive dysfunctions and creating cognitive rehabilitation programs involving the cognotropic pharmacology administration, cognitive training, and the use of transcranial direct current stimulation (tDCS). Cognotropic pharmacology administration aims to enhance cognitive functions contributing to one's longevity [47]. Cognitive training aims to maintain or improve cognitive functions through practicing cognitive tasks [48]. Cognitive training as part of COVID-19 rehabilitation may consist of practicing working memory tasks, taking into account the possible improvement of other functions, such as response inhibition [49]. Unfortunately, the effectiveness of cognitive training is still limited [50]. The use of tDCS in patients with Alzheimer's disease and Parkinson's disease represents an effective cognitive rehabilitation strategy [51].

A possible direction of cognitive rehabilitation is the restoration of unspecific cognitive resources after COVID-19. According to Kahneman [52], higher-order cognitive tasks require the use of unspecific, shared, and exhaustible cognitive ‘resources'. Specific effects on the brain, such as brain fog can be the cause of cognitive resources depletion. Thus, the study of the distribution of cognitive resources in patients with brain fog can play a significant role in the creation of cognitive rehabilitation programs.

The pooled effect size (*d* = −0.35) is close to the result yielded by a meta-analysis of chemobrain in cancer survivors (*d* = −0.26) [16], thus providing another link between these conditions apart from immune and neurological changes [20]. Given that both brain fog and chemobrain are related to a noticeable loss in the quality of life, rehabilitation programs for long COVID could benefit from including EF training already used for cancer survivors.

Effect sizes of a similar magnitude were also yielded for short-term memory in people with long COVID [26]. It brings forth the question on the structure and relationships between different impaired cognitive functions. EF could be at the root of short-term memory deficits, or both could be equally impaired due to cognitive exhaustion caused by fatigue and inflammatory processes.

## 5. Limitations

First, not enough studies fit the criteria. Most studies found by the initial search either utilized a cohort design (no control group) and/or used self-report measures and global test of cognitive functioning (no assessment of EF). Second, there was an inconsistency in the results of the studies yielded by the search: some showed large and significant effect sizes, whereas others found no relationships between the same variables. This could be attributed to the large variation between sample characteristics and measures of EF. Third, it is important to remember that about two thirds of COVID-19 survivors do not report cognitive problems and studies used in the current review selected participants with cognitive complaints and other post-COVID symptoms (e.g., fatigue).

## 6. Conclusions

In this meta-analysis, we have provided evidence of the negative effect of prolonged COVID-19 consequences for executive cognitive functions. Self-reported post-COVID cognitive impairments are usually associated with memory and attention. However, deficits in inhibition, switching, and planning can also be observed in COVID-19 survivors with long-lasting symptoms. This effect was observed in studies comparing post-COVID participants (without previous cognitive dysfunction) and healthy controls on objective measurements of specific EF. The results of the study can specify the picture of brain fog and show its similarity to chemobrain in terms of EF deficiency. The results can contribute to the debates on the matter of post-COVID cognitive impairments for discovering ways of helping people with prolonged consequences of COVID-19.

## Figures and Tables

**Table 1:** Study characteristics and quality assessment.

Study	Design	Location	Demographic characteristics								COVID characteristics		EF measures	Quality score (%)
			Healthy controls				Post-COVID group							
			*n*	nF	Age	Edu	*n*	nF	Age	Edu	Time elapsed	Severity		
Crivelli et al., 2022	Cross-sectional	Argentina	45	20	57 [46–64]	17 [15–18]	45	22	50 [43–63]	17 [15–18]	142 ± 75.9 days	14 (31%) were hospitalized	Trail Making Task-B and Wisconsin Card Sorting Test	100
Guo et al., 2021^a^	Cross-sectional	UK 137 + 130; North America 24+ 33	185	118	[18 ± 61]	28.2% PSE	181	130	[18 ± 61]	63% PSE	3 ± 31 weeks	NA	Wisconsin Card Sorting Test	93
Mattioli et al., 2021	Cross-sectional	Italy	30	22	45.73 [23–62]	18 [8–18]	120	90	47.86 [26–65]	16 [8–18]	12–215 days (mean –125.92 days)	118 mild-moderate; 2 in intensive care unit (ICU)	BACS Tower of London	92
Poletti et al., 2021	Cross-sectional	Italy	165	72	40.57 ± 11.79	13.45 ± 3.79	312	17	52.63 ± 8.81	12.94 ± 3.76	1, 3, and 6 months	86% hospitalized; 4% of them in ICU	BACS Tower of London	100
Wild et al., 2021^a^	Prospective cohort	NA	7832	5539	42.76 ± 14.44	75.61% PSE	478	341	43.41 ± 13.17	82.47% post-sec	3 ± 2 months	15 asymptomatic; 403 mild COVID; 67 hospitalized, 17 in ICU	Tower of London; modified Stroop task (‘double trouble')	93
Zhao et al., 2021^a^	Cross-sectional	NA	46	24	25.03 ± 7.33	NA	36	25	27.71 ± 8.51	NA	167.3 ± 127.9 days	100% mild cases, no hospitalization	Tower of London (version of the original task)	93

Note: ^a^—non-peer-reviewed preprint; nF—number of women; Edu—education in years or % of sample with a degree; PSE—post-secondary education; NA—information not available; EF—executive functions; BACS—brief assessment of cognition in schizophrenia. Data format—mean ± SD [range].

**Table 2:** Measures used in the selected studies.

Measure	Studies	Scores	Description
Wisconsin Card Sorting Test	Guo et al.	Correct, perseveration, and non-perseveration errors reaction times for errors	Contains 60 cards with 1–4 abstract stimuli. Participants match cards according to a rule (matching color, shape, or number of stimuli on the target card and current card). Rules must be inferred from the feedback they get after every attempt. Across 64 trials rules change several times prompting the participants to change their behavior
	Crivelli et al.	Number of categories and perseveration errors	
Tower of London	Mattioli et al.	No. of correct trials	Mattioli et al. and Poletti et al. use the Tower of London task from BACS – Brief Assessment of Cognition in Schizophrenia. The task is to estimate the number of moves between the starting and the target configuration of the three balls. As such, the task requires mental manipulation of the objects
	Poletti et al.		
	Zhao et al.		
	Wild et al.	No. of trials solved within 3 minutes	Wild et al. use a version of the task involving computerized manipulation of the objects that need to be rearranged to reach the target configuration
Trail Making Task-B	Crivelli et al.	Task completion time in seconds	Consists of 25 circles with letters (A–L) and numbers (1–13). Participants match the circles in sequence alternating between numbers and letters
Stroop Test (modified, ‘Double Trouble')	Wild et al.	Total score = no. of correct − No. of errors	Contains congruent, incongruent, and double incongruent trials. The task is to choose a correct word to describe the color of the stimulus word. The task has a time limit of 90 seconds

**Table 3:** Effect sizes from the selected studies tests and/or scales.

Study	Measure	Effect sizes (Cohen's *d*)	Weight	Sample size	SE	Variance	95% CI
Crivelli et al., 2022	TMT	−0.80	20.82	90	0.22	0.05	[−0.37, 1.23]
	WCST—no. of categories	−0.56	21.66	90	0.21	0.05	[−0.98, −0.14]
	WCST—perseverative errors	−0.60	21.52	90	0.22	0.05	[0.18, 1.03]
Guo et al., 2021	WCST—correct answers	0.06	71.64	302	0.12	0.01	[−0.17, 0.29]
	WCST—perseverative errors	0.10	71.59	302	0.12	0.01	[−0.33, 0.13]
	WCST—non perseverative errors	0.01	71.67	302	0.12	0.01	[−0.24, 0.22]
	WCST—RT correct answers	−0.31	70.85	302	0.12	0.01	[0.08, 0.55]
	WCST— RT perseverative errors	−0.17	71.42	302	0.12	0.01	[−0.06, 0.40]
	WCST—non perseverative errors	−0.03	71.67	302	0.12	0.01	[−0.20, 0.26]
Mattioli et al., 2021	TOL	−0.59	23.35	150	0.21	0.04	[−1.00, −0.18]
Poletti et al., 2021	TOL	−0.52	104.69	477	0.10	0.01	[−0.71, −0.33]
Wild et al., 2021	Stroop	−0.41	448.48	8310	0.05	0.00	[−0.50, −0.32]
	TOL	−0.03	450.49	8310	0.05	0.00	[−0.12, 0.06]
Zhao et al., 2021	TOL	−0.25	20.05	82	0.22	0.05	[−0.68, 0.19]

Note: TMT—Trail Making Task-B, WCST—Wisconsin Card Sorting Test, RT—reaction time, TOL—Tower of London, SE—standard error, CI—confidence interval.

## Data Availability

The data supporting this systematic review and meta-analysis are from previously reported studies, which have been cited. The processed data (effect sizes) are included within the article (Table 3).
